# Moderate pediatric burn injury is associated with reduced monocyte HLA-DR expression and heightened whole-blood LPS-induced TNF-α responses

**DOI:** 10.3389/fped.2026.1967322

**Published:** 2026-10-20

**Authors:** Whitman B. Wiggins, Jaimar C. Rincon, Miguel Hernández-Ríos, Valerie Polcz, Christine Rodhouse, Athina L. Yoham, Stefan Richter, Ana Cabral, Lyle L. Moldawer, Philip A. Efron, Robert Maile, Shawn D. Larson

**Affiliations:** 1 Sepsis and Critical Illness Research Center, Department of Surgery, University of Florida College of Medicine, Gainesville, FL, United States; 2 Division of Pediatric Surgery, Department of Surgery, University of Florida College of Medicine, Gainesville, FL, United States

**Keywords:** elispot, HLA-DR, monocytes, pediatric burns, TNF

## Abstract

**Background:**

Burn injury is associated with immune dysregulation and an increased risk of infection and sepsis. In children, however, the immunologic consequences of small and moderate burn injuries remain poorly defined. We compared phenotypic and functional innate immune responses in pediatric patients with small and moderate burn injuries.

**Methods:**

Pediatric patients with small [<15% total body surface area (TBSA); *n* = 14] and moderate (15%–30% TBSA; *n* = 9) burns, along with healthy pediatric controls (*n* = 7), were prospectively enrolled. Whole blood was collected on hospital day 2 and, when feasible, at weekly intervals thereafter. Monocyte HLA-DR expression and circulating myeloid-derived suppressor cell (MDSC) populations were quantified by flow cytometry. Whole-blood innate immune responsiveness was assessed using a TNF-α ELISpot assay following *ex vivo* lipopolysaccharide (LPS) stimulation.

**Results:**

Monocyte HLA-DR expression on hospital day 2 was significantly reduced in patients with moderate burns compared with healthy controls, whereas patients with small burns did not differ significantly from either group. MDSC populations measured one week after injury did not differ among groups. Whole-blood LPS-induced TNF-α responses on hospital day 2 were preserved across all groups and were heightened in patients with moderate burns.

**Conclusions:**

Moderate pediatric burn injury was associated with reduced monocyte HLA-DR expression and heightened acute whole-blood LPS-induced TNF-α responsiveness. Together, these findings suggest that early innate immune alterations after pediatric burn injury reflect concurrent phenotypic and functional dysregulation rather than uniform immune suppression.

## Introduction

Thermal injury is a common cause of pediatric trauma and is associated with substantial long-term physical and psychological morbidity (1–3). Although survival has improved with advances in critical care, pediatric burns continue to impose a major healthcare burden (4–6). Young children are particularly vulnerable because of their exploratory behavior and thinner skin (5, 7). Unlike adults, pediatric burns are most commonly scald injuries and usually involve smaller total body surface area (TBSA) burns (8). Despite this, most burn immunology research has focused on adults and on larger injuries, with findings often extrapolated to pediatric patients.

Burn injury disrupts the body's primary barrier to infection, necessitating reliance on the cellular immune system for host defense (9). The resulting systemic inflammatory response is profound, characterized by elevations in plasma IL-1β, IL-6, and TNF-α (10, 11). Yet, this response coexists with impaired host defense and an increased susceptibility to infection and sepsis (12). Importantly, post-burn immune dysfunction may persist beyond the acute phase; pediatric burn survivors have demonstrated impaired vaccine responses for up to three years after injury (13).

In other forms of critical illness, including sepsis, trauma, and extracorporeal membrane oxygenation, reduced monocyte HLA-DR (mHLA-DR) expression has been associated with immune suppression and poor outcomes (14–16). Myeloid-derived suppressor cells (MDSCs), a heterogeneous population of immature myeloid cells with potent immunosuppressive activity, have also emerged as key mediators of post-injury immune dysfunction (17). Together, mHLA-DR expression and MDSC expansion represent complementary markers of immune competence and dysregulated myeloid responses after critical illness. In adults, MDSC expansion after trauma and sepsis has been associated with impaired antigen presentation, suppression of T-cell activation, prolonged immune dysfunction, and worse clinical outcomes (17, 18). However, the contribution of mHLA-DR expression and MDSC expansion to immune dysfunction in pediatric burn injury remains poorly defined.

We hypothesized that pediatric burn injury would be associated with early immune dysregulation characterized by reduced mHLA-DR expression, expansion of circulating MDSCs, and altered TNF-α responses, with greater abnormalities in moderate burns. To test this, we evaluated the pediatric immune response phenotypically using flow cytometric quantification of monocyte HLA-DR expression and circulating MDSC populations and functionally using ELISpot analysis of whole-blood LPS-induced TNF-α responsiveness. These assays provide complementary information, with mHLA-DR serving as a monocyte-specific phenotypic marker and the ELISpot assessing broader innate immune responsiveness.

## Methods

### Patient recruitment

The study was conducted at the UF Health Shands Children's Hospital pediatric surgery burn service and was approved by the University of Florida Institutional Review Board (IRB202201697). Patients included in this analysis were enrolled between December 2023 and July 2025. Pediatric burn patients were screened and enrolled on the day of admission. Written informed consent was obtained from each subject's parent or legal guardian prior to study participation, and assent was obtained from children 7 years of age and older when appropriate. Eligible participants included pediatric burn patients 6 months to 15 years of age admitted to the pediatric floor or pediatric ICU with either small (<15% TBSA) or moderate (15%–30% TBSA) surface area burns. Burn size was estimated using the Lund-Browder chart by a dedicated burn nurse or surgical resident as part of the pediatric burn service evaluation. The IRB-approved enrollment criteria permitted a ±2 percentage-point tolerance at the upper enrollment threshold, allowing enrollment through 32% TBSA to account for expected variability in clinical TBSA estimation near the 30% cutoff. Healthy controls were defined as non-injured children younger than 16 years undergoing elective pediatric surgery. Control procedures included laparoscopic inguinal hernia repair (*n* = 3), elective cholecystectomy for symptomatic cholelithiasis without evidence of cholecystitis or other biliary pathology (*n* = 2; one laparoscopic and one robot-assisted), bilateral partial mastectomy for gynecomastia (*n* = 1), and elective repair of a small congenital diaphragmatic hernia without associated pulmonary hypertension or chronic lung disease (*n* = 1). Patients were excluded if: informed consent from a parent or legal guardian could not be obtained before blood collection; verbal assent could not be obtained from children older than 7 years; burn size exceeded 32% TBSA; concomitant blunt or penetrating trauma was present; the child had a known malignancy or other immunosuppressive condition, including chronic steroid dependence, inflammatory bowel disease, juvenile arthritis, autoimmune disease, or HIV infection; or the pediatric burn surgery or critical care team determined that blood collection was contraindicated.

### Sample collection

Peripheral blood samples were collected from pediatric participants by venipuncture. In burn-injured patients, samples were obtained on hospital day 2 (±1 day). Children with prolonged hospitalization (>96 h) or those requiring surgical excision of burn wounds had additional samples collected on day 7 (±1 day); depending on their clinical course, additional samples were drawn prior to discharge. Samples from healthy controls were collected once in the operating room following induction of anesthesia and prior to the elective surgical procedure. Blood collection was limited to once weekly and did not exceed the lesser of 50 mL or 3 mL/kg over an 8-week period, in accordance with IRB approval. The reported assay time points were selected based on biological relevance and the availability of sufficient assay-specific samples for group-level comparison. Monocyte HLA-DR and TNF-α ELISpot analyses were performed using hospital day 2 samples to characterize the early immune response, whereas MDSC phenotyping was performed using samples collected approximately one week after injury to assess a later myeloid response. Additional TNF-α ELISpot samples available from three moderate-burn patients at 6–8 weeks were included as a descriptive exploratory analysis. Serial samples were not available from all patients at every time point because repeat collection depended on clinical course and the feasibility of obtaining additional blood samples; consequently, intervening time points did not provide sufficient assay-specific samples for reliable group-level analysis. Assay-specific sample sizes are reported for each analysis.

### *In vitro* LPS-induced TNF-α production capacity by ELISpot

TNF-α-secreting cells were quantified using the human TNF-α Single-Color ELISpot (CTL Inc., OH, USA) in unstimulated and lipopolysaccharide (LPS)-stimulated whole blood, adapted from prior studies (19). Briefly, whole blood was diluted (1:10) and incubated with or without LPS (40 ng/mL) at 37 °C for 24 h. Following incubation, anti-TNF-α antibody solution was added to each well and incubated for 2 h at RT. Streptavidin-AP and blue developer solution were then added and incubated for 15 min in the dark at RT. Plates were scanned using an Immunospot® analyzer with BiosSpot™ Software Suite (CTL Inc., OH, USA).

### Monocyte HLA-DR quantification

HLA-DR expression on circulating CD14^+^ monocytes was analyzed by flow cytometry using the Quantibrite system (Quantibrite™, BD Biosciences, CA, USA). Briefly, 10 µL of anti–human HLA-DR PE (clone L243)/Anti-Monocyte PerCP-Cy5.5 (clone MφP9) was added to 50 µL of whole blood and incubated at RT for 30 min. Subsequently, Buffer EL (Qiagen, MD, USA) was added, and samples were incubated for 10 min to lyse red blood cells. After addition of PBS, samples were centrifuged at 300 × g at RT for 5 min. The supernatant was discarded, and cells were resuspended in 350 µL of PBS. Quantibrite PE Beads (Quantibrite™, BD Biosciences, CA, USA) were prepared according to the manufacturer's instructions, and samples were analyzed on a Bio-Rad ZE5 cell analyzer (Bio-Rad Laboratories, Inc., CA, USA). Monocytes were gated as CD14^+^ cells and BD QuantiBRITE™ PE fluorescent beads were acquired in parallel with blood samples and used to convert the median fluorescence intensity (MFI) results into an antibody bound per cell (ABC) value (20) (Supplementary Figure S1).

### MDSC phenotype analysis

PBMCs were isolated from whole blood by density-gradient centrifugation using Ficoll-Paque PLUS (Cytiva, Global Life Science solutions LLC, DE). The PBMC fraction was collected, washed with PBS and counted using a Cellometer AUTO 2000 (Nexcelom Bioscience LLC, MA). For surface staining, PBMCs (∼1 × 10^6^ cells) were incubated for 15 min at 4 °C with an antibody cocktail targeting MDSC-associated surface markers, including: HLA-DR-APC/Fire 750, CD11b-PE, CD33-FITC, CD14-APC, CD66b-Bv421. Following staining, cells were washed with PBS and acquired on a ZE5 Cell Analyzer (Bio-Rad Laboratories, Hercules, CA). Data were analyzed using FlowJo™ v10.8 (BD Biosciences, Franklin Lakes, NJ). To quantify circulating MDSCs, cells were initially gated on HLA-DR^low/−^CD11b^+^CD33^+^ populations. These were further delineated based on CD14 and CD66b expression to identify specific MDSC subsets: monocytic MDSCs (M-MDSCs: HLA-DR^low/−^CD11b^+^CD33^+^CD14^+^), polymorphonuclear MDSCs (PMN-MDSCs: HLA-DR^low/−^CD11b^+^CD33^+^CD66b^+^), and early MDSCs (E-MDSCs: CD33^+^ CD11b^+^ HLA-DR^−/low^ CD14^−^ CD66b^−^) (21). (Supplementary Figure S2). Compensation was performed using compensation beads (BD Biosciences, Franklin Lakes, NJ). Fluorescence minus one (FMO) samples were included to identify and gate the cells.

### Statistical analysis

Data were analyzed using GraphPad Prism version 10.6.0 (GraphPad Software, San Diego, CA). Given the modest sample size and non-normal distribution of several immune parameters, nonparametric statistical tests were used for group comparisons. Continuous variables were compared using the Kruskal–Wallis test with Dunn's *post hoc* correction for multiple comparisons for analyses involving three independent groups and the Mann–Whitney test for two independent groups, as appropriate. For pairwise comparisons among study groups, Hodges–Lehmann estimates of the between-group location shift with exact confidence intervals were additionally calculated using the Mann–Whitney procedure to describe the magnitude and precision of the observed differences. These estimates and confidence intervals were descriptive and were not adjusted for multiple comparisons; statistical significance for the three-group analyses remained based on the Kruskal–Wallis test with Dunn's correction. Paired comparisons between unstimulated and LPS-stimulated samples from the same subjects were performed using the Wilcoxon matched-pairs signed-rank test. Categorical variables were analyzed using Fisher's exact test. Data are presented as median (range) or *n* (%), as appropriate. A two-sided *p* value <0.05 was considered statistically significant.

## Results

### Patient demographics

Seven healthy controls (HC) undergoing elective pediatric surgery, 14 children with small burns (SB; <15% TBSA), and 9 children with moderate burns (MB; 15%–30% TBSA) were enrolled. Patient demographics and burn characteristics are summarized in Table 1. No statistically significant differences were detected in age, sex, race, ethnicity, or burn mechanism between groups. Nine of 14 patients with small burns required operative debridement and grafting, compared with all patients in the moderate-burn cohort. As shown in Table 2, children with moderate burns underwent a greater number of surgical procedures and had significantly longer hospital and ICU stays. Patients with moderate burns also had numerically greater exposure to enteral nutrition and transfusion, although these differences did not reach statistical significance, whereas inpatient complications were significantly more frequent in the moderate-burn group**.** Despite differences in burn size and resource utilization, all patients were ultimately discharged in stable condition.

**Table 1:** Demographic and clinical characteristics of healthy controls and pediatric burn patients.

Variable	HC *n* = 7	SB (<15% TBSA) *n* = 14	MB (15%-30% TBSA) *n* = 9	*P*-value
Age, years	5.8 (1–14)	7.5 (1.5–15)	6 (1–14)	
Sex, *n* (%)				0.42
Male	5 (71%)	13 (93%)	8 (89%)	
Female	2 (29%)	1 (7%)	1 (11%)	
Race, *n* (%)				0.51
Other	1 (14%)	1 (7%)	2 (22%)	
African American	0	4 (29%)	2 (22%)	
White	6 (86%)	9 (64%)	5 (56%)	
Ethnicity, *n* (%)				0.33
Hispanic	1 (14%)	1 (7%)	3 (33%)	
Non-Hispanic	6 (86%)	13 (93%)	6 (67%)	
TBSA, %	NA	8.1 (1–13)	23 (15–32)	
Burn Mechanism, *n* (%)				0.4
Scald	NA	2 (14%)	3 (33%)	
Flash	NA	3 (21%)	1 (11%)	
Contact	NA	4 (29%)	0 (0%)	
Fire	NA	8 (57%)	5 (56%)	
Burn Depth, *n* (%)				0.99
Partial Thickness (2nd Degree)	NA	9 (64%)	8 (89%)	
Full Thickness (3rd Degree)	NA	8 (57%)	8 (89%)	
Burn Location, *n* (%)				0.83
Head/Neck	NA	4 (29%)	4 (44%)	
Trunk	NA	5 (36%)	8 (89%)	
Upper Extremities	NA	10 (71%)	8 (89%)	
Lower Extremities	NA	7 (50%)	6 (67%)	

Continuous variables are presented as median (range), and categorical variables are shown as *n* (%). *P* values for demographic characteristics represent comparisons among healthy controls (HC), small-burn (SB), and moderate-burn (MB) groups using the Kruskal–Wallis test for continuous variables and Fisher's exact test for categorical variables. TBSA was used to define burn-size groups and was therefore not compared statistically. Moderate burns were defined as 15%–30% TBSA with a prespecified ±2 percentage-point tolerance at the upper enrollment threshold. Burn mechanism, burn depth, and burn location were not mutually exclusive; individual patients could have more than one mechanism, depth, or anatomic location represented, and percentages may therefore sum to greater than 100%.

**Table 2:** In-hospital interventions and outcomes in small- and moderate-burn pediatric patients.

Variable	Small Burn (<15% TBSA, *n* = 14)	Moderate Burn (TBSA 15–30%, *n* = 9)	*P*-value
Required Supplemental Enteral Nutrition, *n* (%)	7 (50%)	8 (89%)	0.09
Required Transfusion, *n* (%)	1 (7%)	4 (44%)	0.06
Required Antibiotics, *n* (%)	2 (14%)	2 (22%)	0.99
Required Surgery, *n* (%)	9 (64%)	9 (100%)	0.12
Number of Surgeries, median (range)	2 (0–5)	4 (1–17)	0.03
Graft/Dermal Substitute, *n* (%)			
Allograft	8 (57%)	8 (89%)	
Autograft	9 (64%)	7 (78%)	
Recell	1 (7%)	3 (33%)	
Patients with ≥1 inpatient complication, *n* (%)	2 (14%)	6 (67%)	0.023
Graft Loss	1 (7%)	5 (56%)	
Wound Infection	0	3 (33%)	
GI Infection	1 (7%)	0	
Bacteremia	0	1 (11%)	
Hospital LOS, days, median (range)	12.5 (3–42)	37 (6–130)	0.002
ICU LOS, days, median (range)	0 (0–22)	11 (0–87)	0.02
Required Mechanical Ventilation, *n* (%)	0 (0%)	2 (22%)	0.14
Ventilator days, among ventilated patients, median (range)	NA	5 (4–6)	

Continuous variables are presented as median (range), and categorical variables are shown as *n* (%). *P* values represent comparisons between small-burn [SB; <15% total body surface area (TBSA)] and moderate-burn (MB; 15%–30% TBSA) patients using the Mann–Whitney *U*-test for continuous variables and Fisher's exact test for categorical variables. Moderate burns were defined as 15%–30% TBSA with a prespecified ±2 percentage-point tolerance at the upper enrollment threshold. Graft/dermal substitute categories and individual inpatient complication types were not mutually exclusive; patients could therefore be represented in more than one category. Statistical significance was defined as *p* < 0.05. GI, gastrointestinal; ICU, intensive care unit; LOS, length of stay; NA, not applicable.

### Burn size-dependent reduction in monocyte HLA-DR (mHLA-DR) expression

mHLA-DR expression was quantified by flow cytometry using whole-blood samples collected on hospital day 2 (T1). mHLA-DR expression was analyzed in healthy controls (HC, *n* = 7), small-burn patients (SB, *n* = 11), and moderate-burn patients (MB, *n* = 6). Median mHLA-DR expression was significantly lower in MB than in HC, whereas SB did not differ significantly from either HC or MB after Dunn's *post hoc* correction (Figure 1). The Hodges–Lehmann estimated shift between MB and HC was −22,939 ABC (exact 96.50% CI, −33,104 to −12,039).

**Figure 1:**
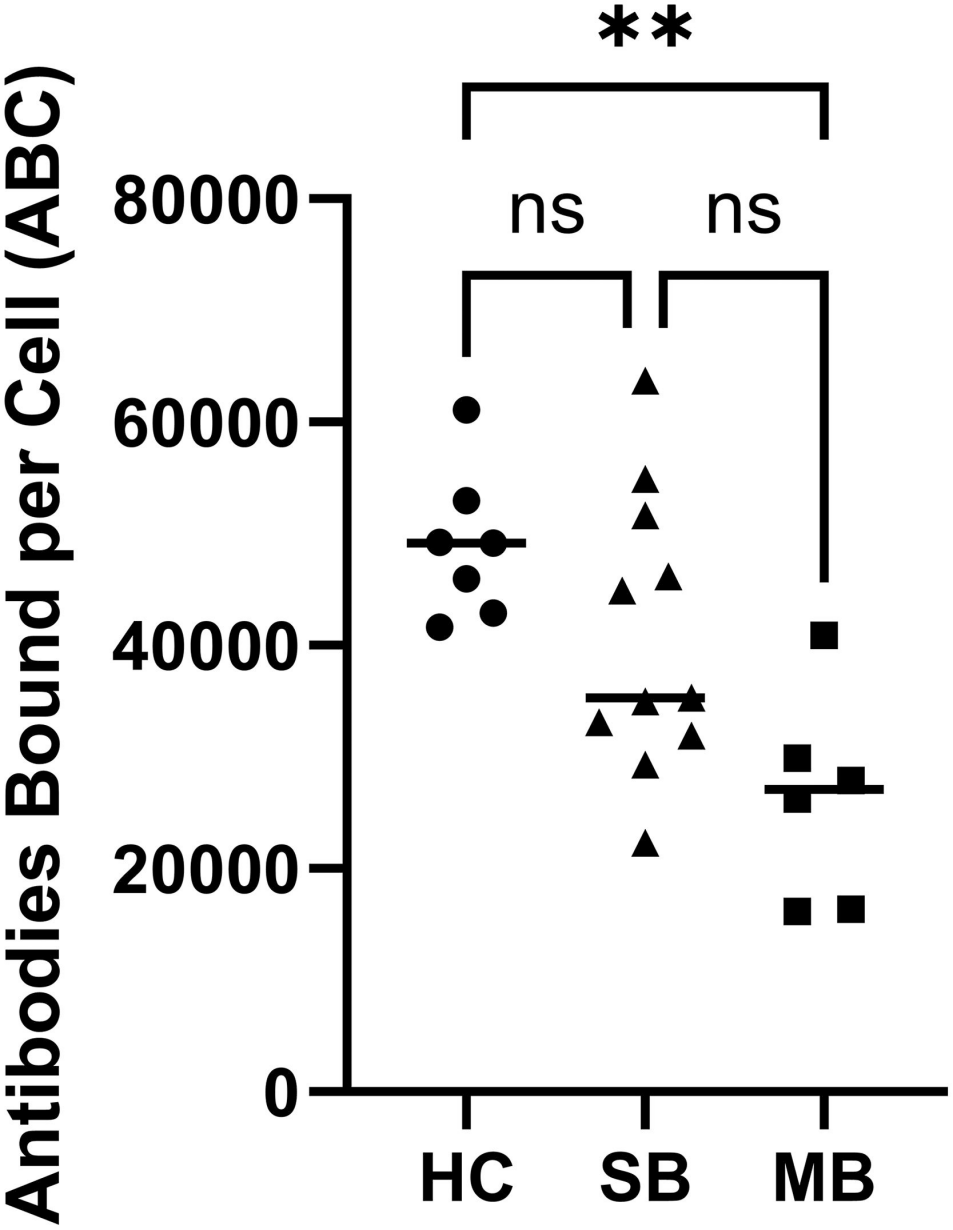
Burn size-dependent reduction in monocyte HLA-DR expression on hospital day 2. Monocyte HLA-DR expression was quantified by flow cytometry in whole-blood samples collected on hospital day 2 and is reported as antibodies bound per cell (ABC). Each symbol represents an individual subject, and horizontal lines indicate the median. HC, healthy controls (*n* = 7); SB, small burns (*n* = 11); MB, moderate burns (*n* = 6). Statistical comparisons were performed using the Kruskal–Wallis test with Dunn's *post hoc* correction. **p < 0.01; ns, not significant.

### MDSC populations are highly variable across all groups

Circulating MDSC populations were quantified by flow cytometry from samples collected one week post-injury in HC (*n* = 7), SB (*n* = 6), and MB (*n* = 6). Total MDSCs were expressed as a percentage of PBMCs, whereas PMN-MDSC, M-MDSC, and E-MDSC subsets were expressed as a percentage of the total MDSC population. Considerable inter-individual variability was observed across all groups. Although PMN-MDSC and M-MDSC percentages appeared higher in the MB group and E-MDSC percentages appeared lower in both burn groups, no statistically significant differences were detected in total MDSC frequency or MDSC subset frequencies among groups (Figure 2). Absolute MDSC counts were also analyzed to account for potential differences in circulating cell abundance. Absolute total MDSC counts were numerically higher in MB compared with HC and SB, but this difference did not reach statistical significance (median [range]: HC, 161,336 [42,282–535,760]; SB, 117,892 [15,810–223,983]; MB, 253,685 [50,531–1,877,920]; *p* = 0.164). Absolute PMN, M-MDSC, and E-MDSC counts were calculated from the absolute total MDSC count and the corresponding subset percentage; no statistically significant differences were detected among groups. Absolute total MDSC counts and subset counts are reported in Supplementary Table S1.

**Figure 2:**
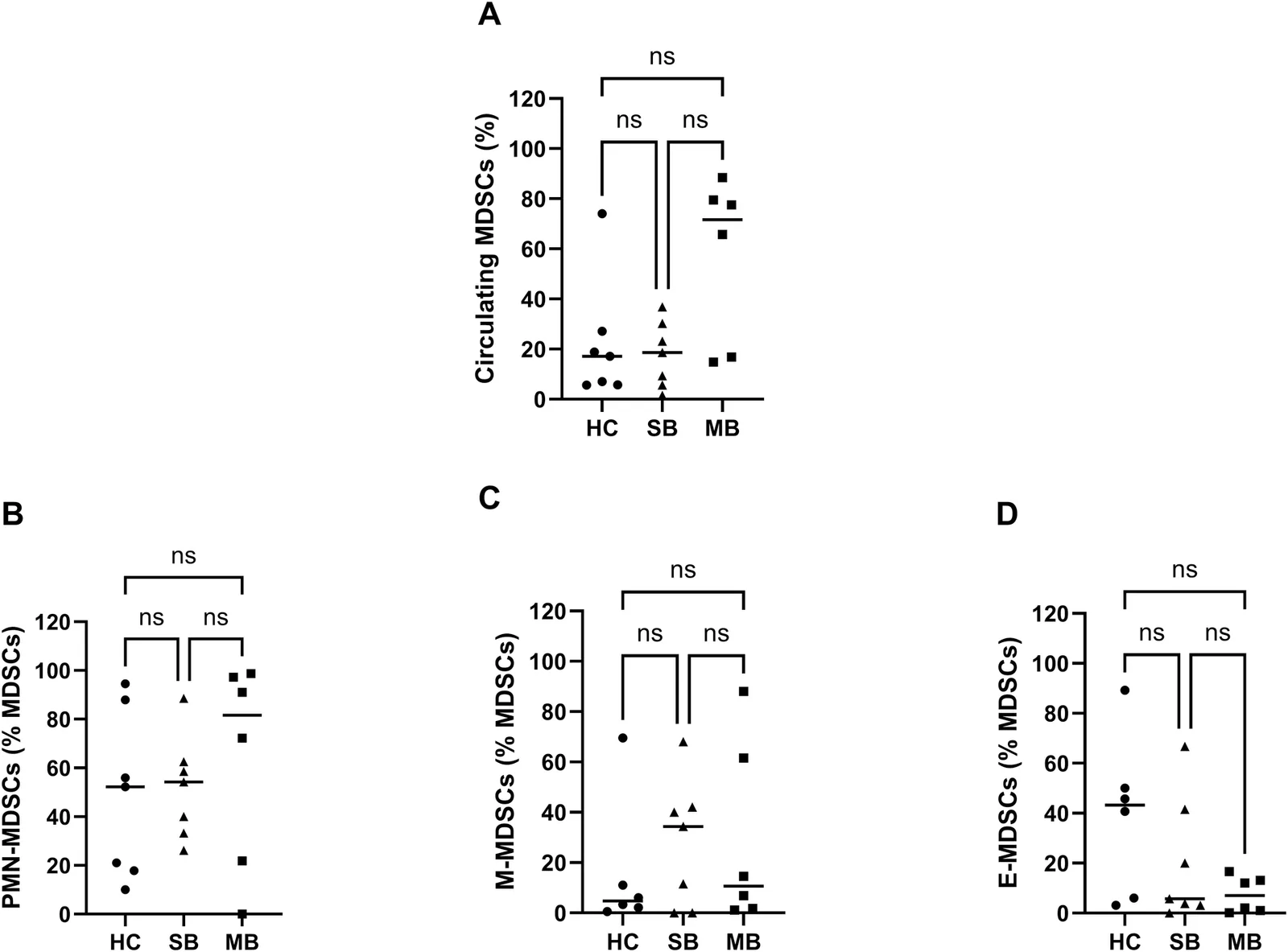
Circulating MDSC populations one week post-injury. Circulating myeloid-derived suppressor cell (MDSC) populations were quantified by flow cytometry in samples collected one week post-injury. **(A)** Total MDSCs are expressed as a percentage of PBMCs. **(B)** PMN-MDSCs, **(C)** M-MDSCs, and **(D)** E-MDSCs are expressed as a percentage of total MDSCs. Each symbol represents an individual subject, and horizontal lines indicate the median. HC, healthy controls (*n* = 7); SB, small burns (*n* = 6); MB, moderate burns (*n* = 6). Statistical comparisons were performed using the Kruskal–Wallis test with Dunn's *post hoc* correction. ns, not significant.

### Whole-blood LPS-induced TNF-α responses are heightened in moderate burns

TNF-α ELISpot responses were quantified in unstimulated and LPS-stimulated whole-blood samples collected on hospital day 2 from HC (*n* = 7), SB (*n* = 7), and MB (*n* = 8). Representative raw ELISpot wells from unstimulated and LPS-stimulated healthy control and moderate-burn samples are shown in Supplementary Figure S3. LPS stimulation significantly increased SFU, SS, and TE within each cohort compared with unstimulated samples (*p* < 0.01; Figure 3), demonstrating preserved LPS responsiveness across all groups. Among LPS-stimulated samples, responses were greatest in the moderate-burn cohort: SFU, SS, and TE were significantly higher in MB than HC (*p* < 0.05), and TE was also significantly higher in MB than SB (*p* < 0.05). The corresponding Hodges–Lehmann estimated MB–HC shifts were 294 for SFU (exact 95.99% CI, 36–506), 1.203 for SS (exact 95.99% CI, 0.3966–2.521), and 2,503 for TE (exact 95.99% CI, 974.7–3,686); the estimated MB–SB shift in TE was 2,229 (exact 95.99% CI, 403.2–3,473). SB did not differ significantly from HC (Figure 4).

**Figure 3:**
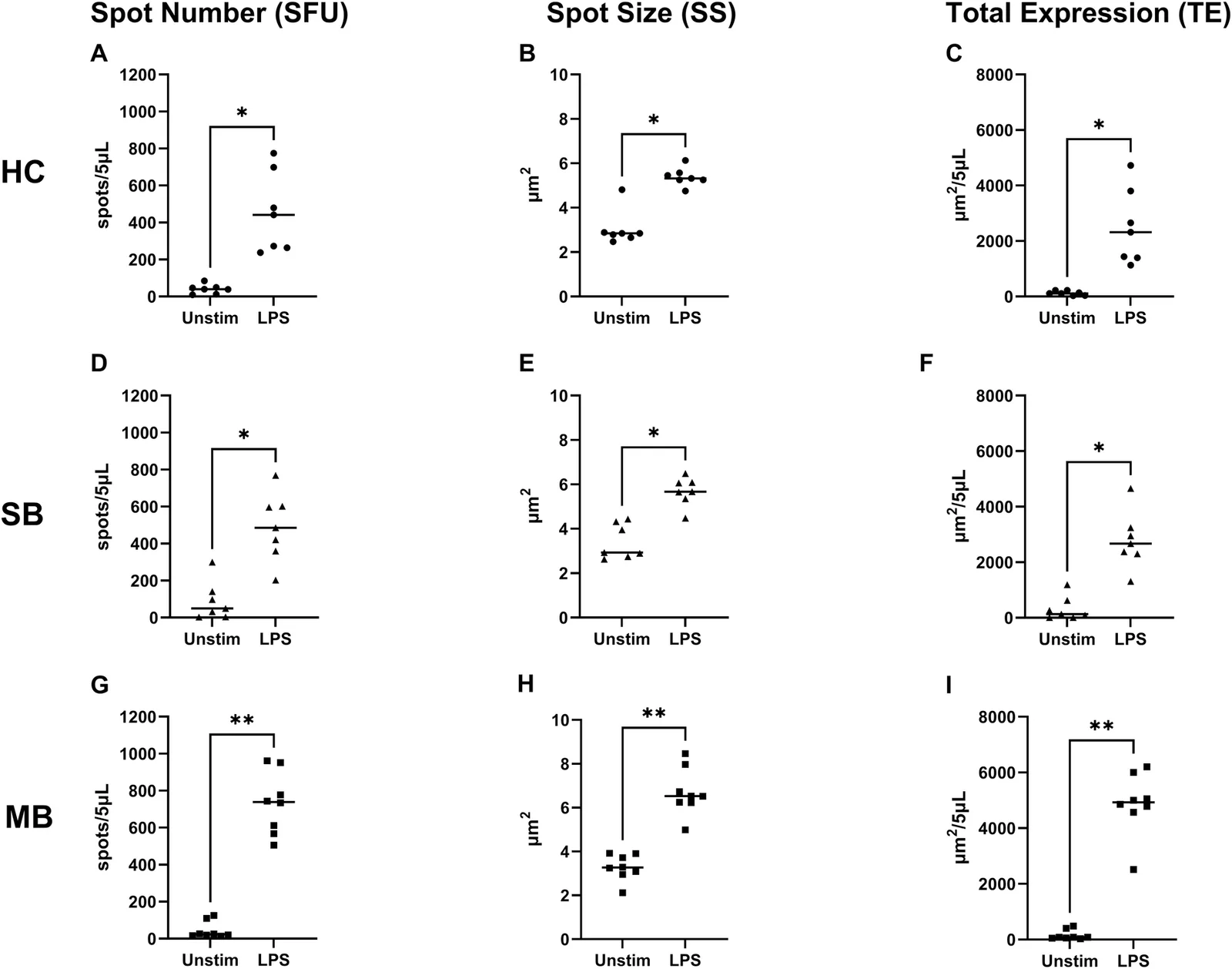
Day 2 TNF-α ELISpot responses under unstimulated and LPS-stimulated conditions. TNF-α production was quantified by ELISpot analysis of whole-blood samples collected on hospital day 2 under unstimulated and lipopolysaccharide (LPS)-stimulated conditions. Spot number (SFU), spot size (SS), and total expression (TE) are shown for healthy controls [HC; **(A–C)**], small burns [SB; **(D–F)**], and moderate burns [MB; **(G–I)**]. Each symbol represents an individual subject, and horizontal lines indicate the median. HC, healthy controls (*n* = 7); SB, small burns (*n* = 7); MB, moderate burns (*n* = 8). Statistical comparisons between paired unstimulated and LPS-stimulated samples were performed using the Wilcoxon matched-pairs signed-rank test. *p < 0.05, **p < 0.01.

**Figure 4:**
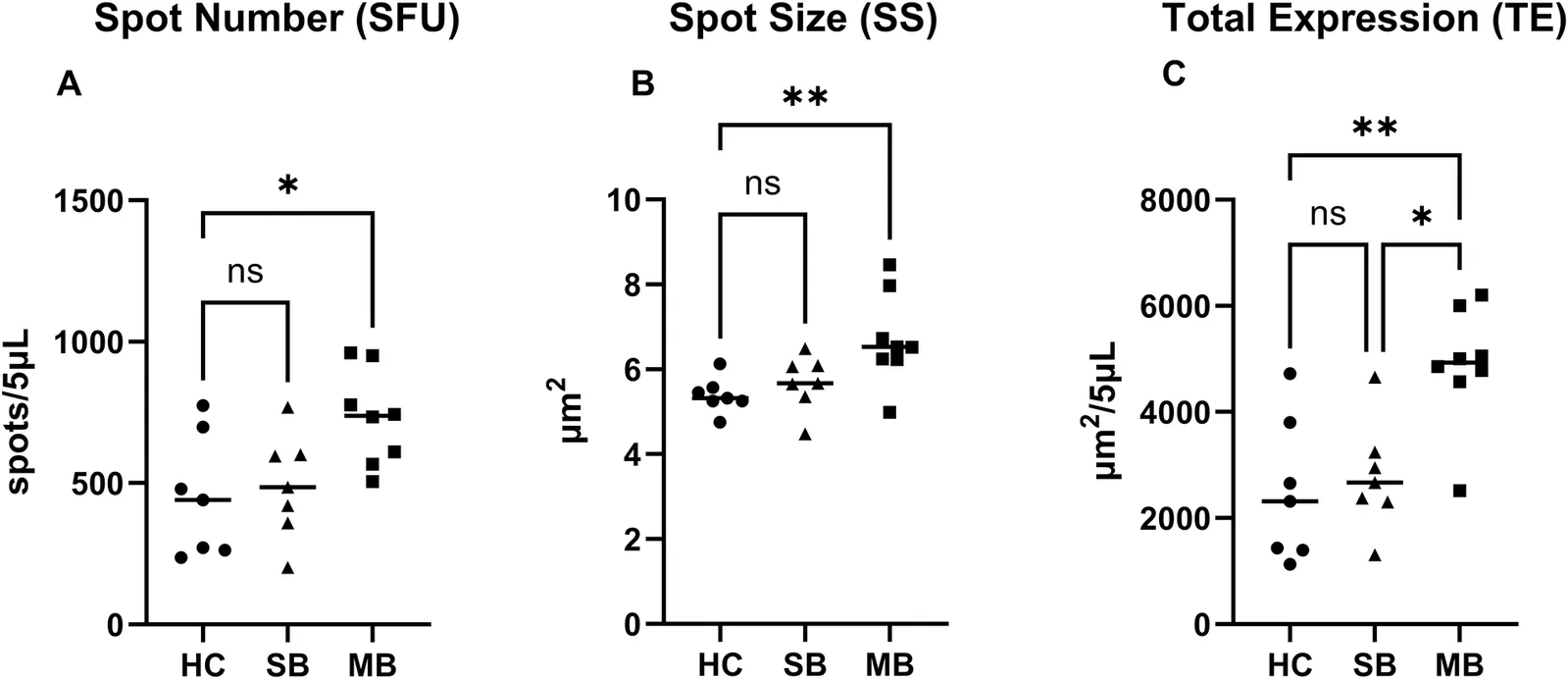
Day 2 TNF-α ELISpot responses in LPS-stimulated samples across study groups. TNF-α production was quantified by ELISpot analysis of LPS-stimulated whole-blood samples collected on hospital day 2. Spot number [SFU; **(A)**], spot size [SS; **(B)**], and total expression [TE; **(C)**] are shown for healthy controls (HC), small burns (SB), and moderate burns (MB). Each symbol represents an individual subject, and horizontal lines indicate the median. HC, healthy controls (*n* = 7); SB, small burns (*n* = 7); MB, moderate burns (*n* = 8). Statistical comparisons were performed using the Kruskal–Wallis test with Dunn's *post hoc* correction. *p < 0.05, **p < 0.01; ns, not significant.

In an exploratory analysis, whole-blood LPS-induced TNF-α responses were evaluated in samples collected 6–8 weeks after burn injury from three patients with moderate burns. Compared with acute samples collected on hospital day 2 (*n* = 8), samples collected at 6–8 weeks (*n* = 3) showed lower SFU and TE, whereas SS appeared similar. Healthy controls (*n* = 7) are shown for reference (Supplementary Figure S4). Given the limited number of late samples, these data were considered hypothesis-generating and were presented descriptively without inferential statistical testing.

### Patients with clinical complications demonstrate lower monocyte HLA-DR expression

In an exploratory analysis of burn patients with available immune-assay data, patients with inpatient complications, defined as graft loss, wound infection, gastrointestinal infection, and bacteremia, had greater burn size than those without inpatient complications [*n* = 8 vs. *n* = 10; median [range]: 25% [10%–32%] vs. 11% [2%–22%] TBSA; *p* = 0.0348]. Among patients with available day 2 mHLA-DR data, those with complications had lower monocyte HLA-DR expression than those without complications [*n* = 6 vs. *n* = 9; median [range]: 28,603 [16,140–33,104] vs. 40,910 [22,325–63,675] ABC; *p* = 0.0360]. In contrast, whole-blood LPS-induced TNF-α ELISpot responses did not differ significantly between complication groups for SFU, SS, or TE **(**Figures 5,6).

**Figure 5:**
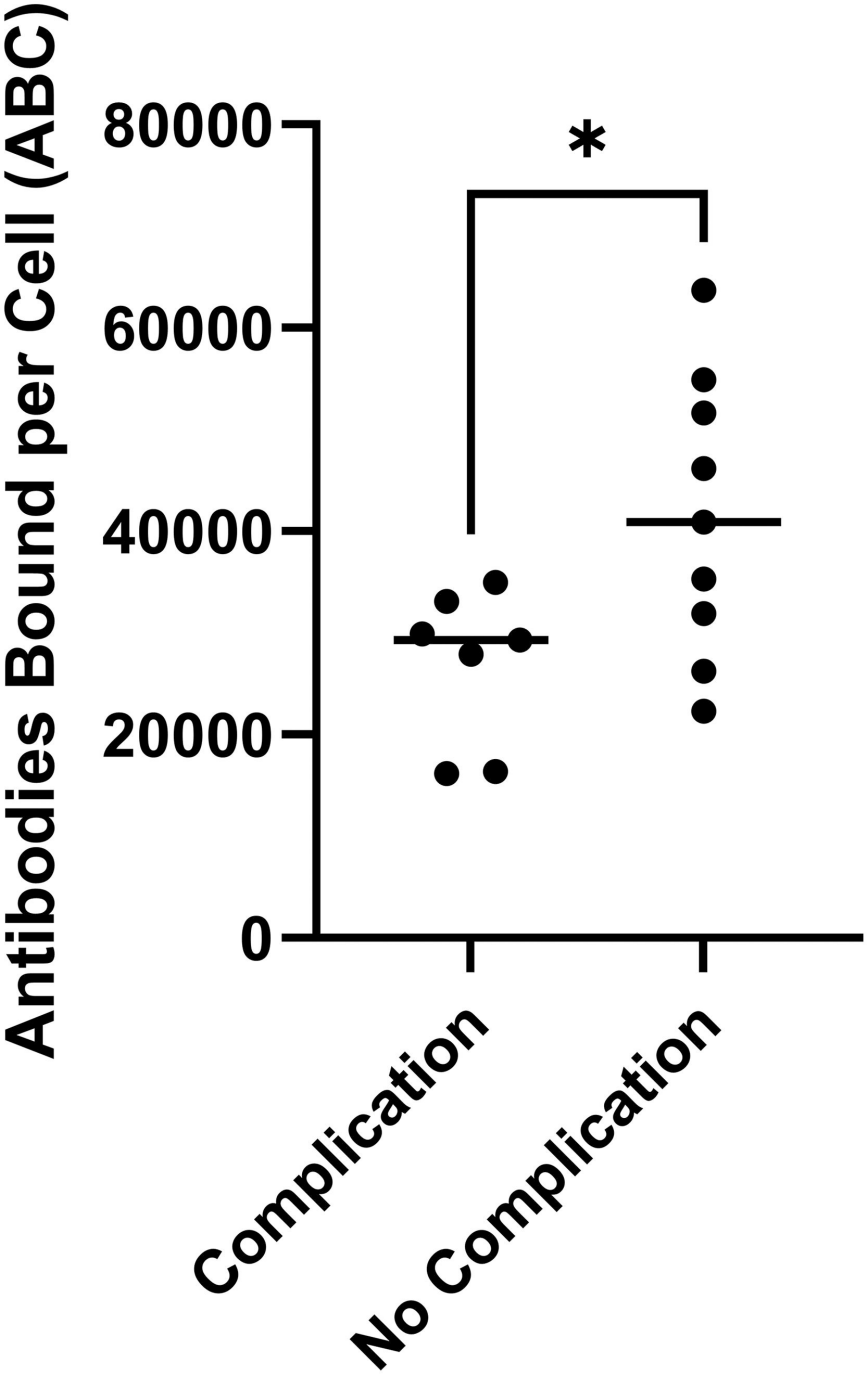
Inpatient complications are associated with lower monocyte HLA-DR expression. Monocyte HLA-DR expression was compared between small- and moderate-burn patients with inpatient complications (*n* = 6) and those without inpatient complications (*n* = 9). Inpatient complications were defined as graft loss, wound infection, gastrointestinal infection, and/or bacteremia. Monocyte HLA-DR expression was quantified by flow cytometry in whole-blood samples collected on hospital day 2 and is reported as antibodies bound per cell (ABC). Each symbol represents an individual subject, and horizontal lines indicate the median. Statistical comparison was performed using the Mann–Whitney test. *p < 0.05.

**Figure 6:**
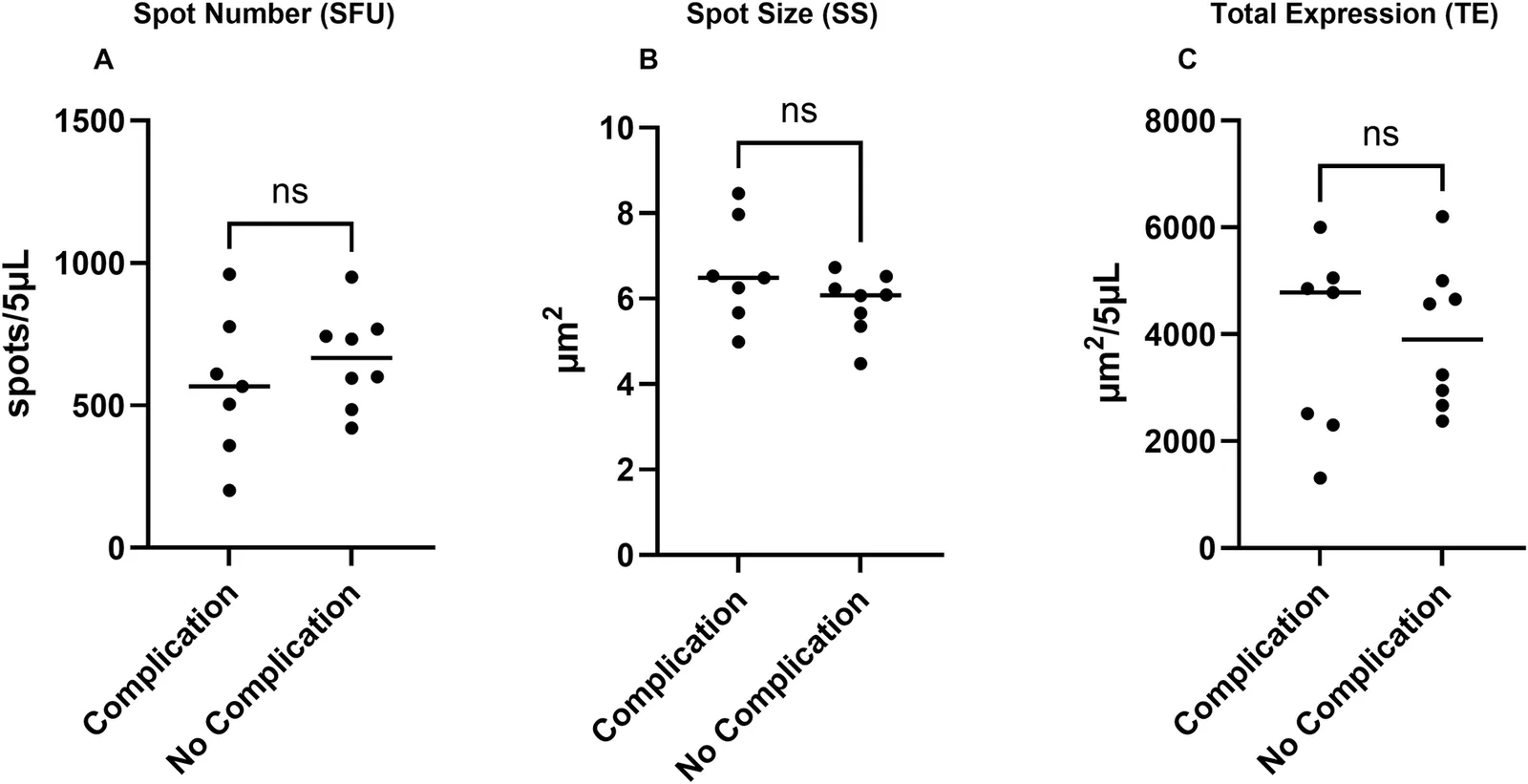
LPS-stimulated TNF-α ELISpot responses do not differ by inpatient complication status. LPS-stimulated TNF-α production was compared between small- and moderate-burn patients with inpatient complications (*n* = 7) and without inpatient complications (*n* = 8). Inpatient complications were defined as graft loss, wound infection, gastrointestinal infection, and/or bacteremia. Spot-forming units [SFU; **(A)**], spot size [SS; **(B)**], and total expression [TE; **(C)**] were measured by ELISpot assay. Each symbol represents an individual subject, and horizontal lines indicate the median. Statistical comparisons were performed using the Mann–Whitney test. ns, not significant.

## Discussion

Severe burn injury induces significant immune dysregulation that contributes to infectious complications, sepsis, and delayed wound healing (22). Concurrent pro-inflammatory and immunosuppressive responses have been described after severe burn injury, including in adult cohorts, and in other forms of critical illness (22–24). Although the immune response to burn injury has been extensively studied in adults, pediatric data remain more limited, particularly regarding differences in immune responses across burn-size groups (8). In this study, moderate pediatric burns were associated with reduced monocyte HLA-DR expression alongside heightened acute whole-blood LPS-induced TNF-α responsiveness, demonstrating concurrent alterations across complementary phenotypic and functional immune domains.

Monocyte HLA-DR expression is a well-established phenotypic marker associated with monocyte immune competence, with reduced expression observed in states of immune dysfunction (25). In adults, diminished HLA-DR expression has consistently been observed following sepsis, major trauma, and severe burn injury and may persist in patients who subsequently develop nosocomial infections (23, 25, 26). Our findings are consistent with this literature, with reduced monocyte HLA-DR expression observed in children with moderate burns. Although HLA-DR expression in children with small burns did not differ significantly from healthy controls at the group level, considerable inter-individual variability was observed, with some patients demonstrating values overlapping the moderate-burn group and others more closely resembling healthy controls. This heterogeneity suggests that monocyte deactivation may not occur uniformly after smaller burn injuries and that burn size alone may not fully account for differences in monocyte HLA-DR expression. Larger cohorts will be needed to determine the clinical significance of this variability.

The mechanisms underlying burn-induced monocyte deactivation are likely multifactorial. Severe thermal injury triggers the release of damage-associated molecular patterns (DAMPs), stress hormones, and catecholamines, all of which can influence monocyte function (27). Recent pediatric burn work has also linked early alterations in DAMP-associated proteins, FAS(APO), and AKT/mTOR phosphoprotein signaling with subsequent nosocomial infection (28). Elevated cortisol and catecholamine levels in the early post-burn period have been shown to suppress MHC class II expression and impair cytokine signaling, contributing to transient immunosuppression (29). In parallel, persistent systemic inflammation and myeloid activation may promote compensatory anti-inflammatory signaling, including increased IL-10, which may further contribute to monocyte dysfunction (30).

In contrast to the HLA-DR findings, circulating MDSC populations did not differ significantly between groups at one-week post injury. MDSCs are a heterogeneous population of immature myeloid cells with immunosuppressive capacity that expand following severe infection, trauma, and other forms of critical illness, where they have been linked to impaired T-cell function, chronic critical illness, and increased risk of nosocomial infection (31–33). Because severe injury can drive dysregulated myelopoiesis and pathologic myeloid activation, MDSCs represented a biologically plausible contributor to post-burn immune dysfunction (34, 35). However, in this pediatric cohort, both total and subset MDSC frequencies demonstrated substantial variability across healthy controls, small burns, and moderate burns, and no consistent difference was observed between groups. Notably, a subset of patients with moderate burns demonstrated relatively high circulating MDSC frequencies despite the absence of a significant group-level difference. This pattern further highlights the inter-individual heterogeneity of the post-burn myeloid response and warrants further investigation in larger cohorts. Such variation may reflect differences in the timing and magnitude of MDSC recruitment after injury, age-dependent differences in pediatric MDSC biology, or limited power to detect smaller differences in this cohort (36). The one-week sampling time point may also have limited our ability to characterize MDSC kinetics, as prior work has shown that MDSC phenotype and function can evolve over time during critical illness (37, 38). Because MDSCs were assessed approximately one week after injury, these findings reflect a later phase of the post-burn immune response than the day 2 mHLA-DR and ELISpot measurements. Thus, although these data do not demonstrate a clear difference in circulating MDSC populations at one week following pediatric burn injury, they do not exclude a role for MDSCs in evolving post-burn immune dysfunction. Larger studies with serial sampling are needed to better define the temporal kinetics and immunologic significance of MDSC activation in this population.

Functional assessment using the TNF-α ELISpot assay provided complementary insight into innate immune responsiveness following burn injury. LPS stimulation produced robust TNF-α responses across all groups, with the greatest responses observed in the moderate-burn cohort. This heightened early response may reflect broader activation of the innate immune compartment after more extensive burn injury, consistent with prior evidence of early inflammatory signaling following thermal injury (27). Importantly, monocyte HLA-DR expression and whole-blood LPS-induced TNF-α responses assess distinct but complementary immune domains: monocyte HLA-DR expression is a phenotypic marker associated with monocyte immune competence, whereas the whole-blood ELISpot assay captures an integrated functional response across multiple innate immune cell populations rather than a single cellular compartment. Although neutrophils are numerically predominant in whole blood, prior studies indicate that monocytes and other mononuclear cells are major contributors to LPS-induced TNF-α production, whereas neutrophils have substantially lower *de novo* TNF-α-producing capacity on a per-cell basis (39, 40). Accordingly, these assays were not intended to identify a shared cellular mechanism or to imply that changes in monocyte HLA-DR should parallel whole-blood TNF-α production. The coexistence of reduced monocyte HLA-DR expression and heightened whole-blood LPS-induced TNF-α responses therefore supports concurrent alterations across complementary domains of innate immunity.

In an exploratory serial analysis, whole-blood LPS-induced TNF-α responses were lower in samples collected 6–8 weeks after injury than in acute moderate-burn samples. Spot number and total expression were lower, whereas spot size was similar. Similar changes in immune responsiveness over time have been described after adult burn injury and sepsis (24, 41). However, because later samples were available from only three patients, these findings should be considered preliminary and hypothesis generating. Larger studies with paired serial sampling are needed to determine whether innate immune responsiveness changes during recovery from pediatric burn injury.

Although the measured immune endpoints in children with small burns did not differ significantly from healthy controls at the group level, prior work suggests that non-severe pediatric burns are not immunologically inert, with long-term immune dysfunction and diminished vaccine responses reported years after injury (13). Recent pediatric burn studies have also linked immune alterations to adverse outcomes. Penatzer et al. grouped patients according to subsequent nosocomial infection status and found that children who later developed infection had lower early monocyte HLA-DR expression and reduced *ex vivo* LPS-induced TNF-α responses. However, patients who subsequently developed infection also had greater burn severity across several correlated measures, including larger TBSA burns, more full-thickness injury, and more inhalational injury, making it difficult to determine the independent contribution of any single aspect of injury severity to the observed reduction in HLA-DR expression (42).

In an exploratory analysis of our cohort, patients with inpatient complications, defined as graft loss, wound infection, gastrointestinal infection, and/or bacteremia, also had greater burn size than those without complications. These patients demonstrated lower day 2 monocyte HLA-DR expression, whereas LPS-stimulated TNF-α ELISpot responses did not differ by complication status. Although this analysis is limited by small sample size and confounding by burn size, the observed association between lower HLA-DR expression and complications is directionally consistent with prior work linking monocyte deactivation to adverse outcomes after burn injury (23, 42).

Taken together, our findings indicate that acute whole-blood LPS-induced TNF-α responsiveness does not decline in parallel with monocyte HLA-DR expression after moderate pediatric burn injury. This mixed innate immune phenotype, characterized by reduced monocyte HLA-DR expression alongside heightened inducible innate cytokine responsiveness, was observed at day 2 in the moderate-burn group.

Limitations of this study include the modest sample size and single-center design, which limit generalizability. Given the small assay-specific sample sizes, this study is exploratory and underpowered to detect modest between-group differences; therefore, nonsignificant findings should not be interpreted as evidence of equivalence between groups. The cohort was predominantly male, particularly among burn patients, limiting our ability to assess potential sex-associated differences in post-burn immune responses. Given recognized sex-related differences in immune responses and reported differences in outcomes following burn injury (43–45), the limited representation of female patients may have influenced the observed immune profiles and may limit the generalizability of our findings across sexes. Demographic representation was also not fully balanced across groups; although race did not differ statistically, no healthy controls were African American, compared with 29% of the small-burn cohort and 22% of the moderate-burn cohort. These demographic imbalances may limit generalizability and represent a potential source of residual confounding. The healthy control cohort consisted of children undergoing elective surgical procedures, and heterogeneity in underlying surgical diagnoses may also have contributed to variability in immune measurements. Although anesthesia or sedation exposure was common across the study population, differences in peri-procedural exposures between groups cannot be completely excluded as an additional source of variability. Clinical interventions also differed with burn severity, and perioperative and supportive-care exposures, including operative treatment, transfusion, antibiotics, analgesic medications, and nutritional support, varied across patients. Because these interventions are closely linked to injury severity, the present study was not designed or adequately powered to distinguish their independent effects from those associated with the burn injury and its clinical course. Many patients had mixed-depth injuries, limiting meaningful stratification by burn depth in this modest cohort. Burn depth may provide additional biological information beyond TBSA, and larger studies incorporating quantitative measures of burn depth will be needed to evaluate its contribution to post-burn immune responses. This study was designed as a descriptive immune phenotyping study and was not powered to determine associations between immune markers and infection prevalence, organ injury, or other clinical outcomes. Although inpatient complications were recorded, detailed adjudication of infectious complications, including pneumonia, urinary tract infection, and other site-specific infections, was limited by the small number of events. Inter-individual variability, particularly in the MDSC analyses, may also have obscured smaller but biologically meaningful differences. Treg populations were not assessed, limiting evaluation of additional suppressive immune mechanisms that may interact with MDSC biology after burn injury. The TNF-α ELISpot assay provides a focused assessment of whole-blood LPS-induced TNF-α responsiveness and does not identify the cellular source of TNF-α. Standardized leukocyte subset counts were not systematically collected, limiting our ability to determine the extent to which differences in whole-blood TNF-α responses may reflect differences in cellular composition. It also does not capture the broader circulating inflammatory or anti-inflammatory cytokine milieu. Longitudinal sampling was limited and was not available across all assays. Later ELISpot samples were available from only three moderate-burn patients; therefore, these exploratory observations cannot establish a longitudinal trajectory and should be interpreted cautiously. Broader inflammatory profiling was not systematically performed; CRP, IL-6, and IL-10 were not consistently collected, and additional functional leukocyte assays, including phagocytosis, reactive oxygen species production, and cellular migration, were not performed. Despite these limitations, this study provides an integrated phenotypic and functional assessment of immune responses after small and moderate pediatric burn injury and offers a useful framework for larger studies incorporating serial immune phenotyping, functional leukocyte assays, standardized organ injury measures, Treg characterization, comprehensive cytokine profiling, and detailed infectious outcome adjudication.

## Conclusion

Moderate pediatric burn injury was associated with reduced monocyte HLA-DR expression and heightened acute whole-blood LPS-induced TNF-α responsiveness, whereas small burns did not differ significantly from healthy controls in this cohort. These complementary phenotypic and functional findings suggest that early post-burn immune dysfunction in children is not characterized by uniform immune suppression but instead reflects a mixed innate immune phenotype that varies with burn severity.

## Data Availability

The raw data supporting the conclusions of this article will be made available by the authors, without undue reservation.
