# Supplementary material for: Moderate pediatric burn injury is associated with reduced monocyte HLA-DR expression and heightened whole-blood LPS-induced TNF-α responses
Source: Front Pediatr. 2026 Oct 20;14:1967322. doi: 10.3389/fped.2026.1967322 (PMC13727738; doi:10.3389/fped.2026.1967322)
Supplement: Supplementary file 1 [file Supplementaryfile1.docx]

**Supplementary Figure 1. Gating strategy for monocyte HLA-DR quantification.**
Whole blood was analyzed by flow cytometry to quantify HLA-DR expression on circulating monocytes. (A) Cells were first identified based on forward and side scatter characteristics. (B) Monocytes were gated as CD14+ cells. (C) HLA-DR expression was quantified within the CD14+ monocyte population using a PE-conjugated anti–HLA-DR antibody. (D–E) Quantibrite PE beads were used to convert fluorescence intensity to antibodies bound per cell (ABC).

**Supplementary Figure 2. Gating strategy for the identification of circulating MDSC subsets.** Doublets were excluded and live PBMCs were gated (A-C). To quantify MDSCs, flow cytometry was performed on fresh PBMCs. MDSCs were identified as (D) HLA-DR-/low, (E) CD11b+CD33+ cells with (F) PMN-MDSCs, M-MDSCs, and E-MDSCs subsets based on expression CD66b and CD14, respectively.

**Supplementary Figure 3. Representative TNF-α ELISpot wells from healthy control and moderate-burn samples.** Representative ELISpot wells are shown from one healthy control and one moderate-burn subject under unstimulated and LPS-stimulated conditions. Wells I–II show unstimulated healthy control replicates, wells III–IV show unstimulated moderate-burn replicates, wells V–VI show LPS-stimulated healthy control replicates, and wells VII–VIII show LPS-stimulated moderate-burn replicates.

**Supplementary Figure 4. Acute and late whole-blood LPS-induced TNF-α ELISpot responses in moderate burns.**

TNF-α production was quantified by ELISpot analysis of LPS-stimulated whole-blood samples. Spot number (SFU; A), spot size (SS; B), and total expression (TE; C) are shown for healthy controls (HC; n=7), acute moderate-burn samples collected on hospital day 2 (Acute; n=8), and late moderate-burn samples collected 6–8 weeks after injury (Late; n=3). Each symbol represents an individual subject, and horizontal lines indicate the median. Healthy controls are shown for reference. Given the limited number of late samples, these data are considered hypothesis-generating and are presented descriptively without inferential statistical testing. Total expression in panel C is displayed on the original scale, consistent with Figure 4C.

**Supplementary Table 1. PBMC-derived absolute MDSC and MDSC subset counts at one week post-injury.**

| **Variable** | **Healthy Controls (n=7)** | **Small Burns**  **(n=6)** | **Moderate Burns (n=6)** | **p-value** |
| --- | --- | --- | --- | --- |
| **Total MDSCs** | 161,336  (42,282–535,760) | 117,892  (15,810–223,983) | 253,685  (50,531–1,877,920) | 0.164 |
| **PMN-MDSCs** | 29,431  (8,879–506,829) | 58,974  (6,324–169,716) | 141,604  (0–1,710,785) | 0.538 |
| **M-MDSCs** | 7,761  (1,018–112,290) | 14,189  (0–66,929) | 73,551  (2,780–239,144) | 0.289 |
| **E-MDSCs** | 21,287  (5,507–260,220) | 4,957  (0–92,953) | 21,834  (283–38,804) | 0.415 |

Absolute total MDSC counts were calculated from total PBMC counts and total MDSC frequency. Absolute PMN-MDSC, M-MDSC, and E-MDSC counts were calculated by multiplying the absolute total MDSC count by the corresponding subset percentage. Values are presented as median (range). Overall p-values were calculated using Kruskal–Wallis testing. MDSC, myeloid-derived suppressor cells.
